# Inhaler Devices as Potential Bacterial Reservoirs: A 30-Day Microbiota Analysis in Non-Cystic Fibrosis Bronchiectasis

**DOI:** 10.3390/biomedicines14092132

**Published:** 2026-09-21

**Authors:** Jiyeon Baek, Ok Joo Sul, Hye Won Choi, Chaeyeong Jung, Sunju Lee, Jinkyeong Park, Harim Koo, Byungsuk Kwon, Fernando Sergio Leitao Filho, Seung Won Ra

**Affiliations:** 1 Medical Science, Asan Medical Institute of Convergence Science and Technology, Asan Medical Center, University of Ulsan College of Medicine, Seoul 05505, Republic of Korea; 2 Biomedical Research Center, Ulsan University Hospital, School of Medicine, University of Ulsan College of Medicine, Ulsan 44033, Republic of Korea; 3 Department of Medicine, Soonchunhyang University College of Medicine, Cheonan 31151, Republic of Korea; 4 School of Medical Science, University of Ulsan, Ulsan 44610, Republic of Korea; 5 Biomedical Research Institute, Ulsan University Hospital, Ulsan 44033, Republic of Korea; 6 Department of Pulmonary, Allergy and Critical Care Medicine, Kyung Hee University Hospital at Gangdong, Seoul 05278, Republic of Korea; 7 Department of Medical Science Convergence, Graduate School of Medical Science, Basic-Clinical Convergence Research Institute, University of Ulsan, Ulsan 44033, Republic of Korea; 8 School of Biological Sciences, University of Ulsan, Ulsan 44610, Republic of Korea; 9 Division of Pulmonology, Department of Internal Medicine, Universidade Federal de São Paulo, São Paulo 04023-062, Brazil; 10 Evidence-Based Medicine and Urgent Medicine Unit, Department of Internal Medicine, Universidade Federal de São Paulo, São Paulo 04023-062, Brazil; 11 Division of Pulmonology, Department of Internal Medicine, Ulsan University Hospital, University of Ulsan College of Medicine, Ulsan 44033, Republic of Korea

**Keywords:** inhaler devices, bronchiectasis, microbiome, microbial dysbiosis, *Pseudomonas aeruginosa*, pressurized metered-dose inhaler, Respimat^®^

## Abstract

**Background/Objectives:** Handheld inhalers are essential for the management of bronchiectasis, yet their role as potential microbial reservoirs remains poorly understood. We aimed to characterize and compare the bacterial communities in pressurized metered-dose inhalers (pMDIs) and Respimat^®^ devices after 30 days of clinical use. **Methods:** In this 30-day prospective study of eight adults with non-cystic fibrosis bronchiectasis, inhaler devices (six pMDIs and four Respimat^®^ devices) were analyzed using 16S rRNA gene sequencing of the V4 region. Total bacterial loads (quantitative PCR), α- and β-diversity, differential taxon abundance (ANCOM-BC), and microbial source tracking (SourceTracker2) were compared between device types. **Results:** After 30 days, pMDIs showed significantly higher bacterial loads than negative controls (adjusted *p* < 0.05), whereas Respimat^®^ loads were indistinguishable from those of controls. Compared to Respimat^®^ devices, pMDIs showed a trend toward microbial simplification (α-diversity: Shannon index, *p* = 0.08). However, overall community structures did not differ significantly (β-diversity: PERMANOVA, adjusted *p* = 0.18). ANCOM-BC identified 12 amplicon sequence variants (ASVs) significantly enriched in pMDIs, including one belonging to the genus *Pseudomonas*, compared to only one ASV in Respimat^®^ devices. Paired device analyses between two participants who used both devices also revealed differences in the relative abundance of specific potentially pathogenic genera. For instance, in one participant, the pMDI was dominated by *Staphylococcus* (70.9% vs. 2.5% in the paired Respimat^®^). **Conclusions:** After 30 days of use by patients with bronchiectasis, bacterial DNA was detected in the liquid contents of both inhaler types. Only pMDIs showed a significant increase in total bacterial load relative to negative controls, and pMDIs harboured more differentially abundant ASVs than Respimat^®^ devices, suggesting that pMDIs may be more susceptible to microbial contamination in patients with non-cystic fibrosis bronchiectasis.

## 1. Introduction

Pressurized metered-dose inhalers (pMDIs) and Respimat^®^ inhalers (Boehringer Ingelheim International GmbH, Ingelheim, Germany) are the primary delivery systems for patients with chronic airway diseases [1,2]. These devices differ significantly in terms of their internal architecture and drug delivery mechanisms. Respimat^®^ features a unique uniblock structure, utilizing a nozzle system with colliding liquid jets and an integrated non-return valve to ensure a uniform, fine-particle mist [3]. In contrast, pMDI consists of a canister, a metering valve, and a nozzle actuator. Conventional pMDIs rely on a “press-and-breathe” mechanism; when the canister is pushed against the actuator to release the drug-propellant mixture, the internal components are briefly but significantly exposed to the external environment [4].

Several culture-based studies have highlighted the potential for microbial contamination associated with routine inhaler use [5,6,7]. For example, Borovina et al. found that 80.0% of 45 used pMDI mouthpieces were colonized with *Staphylococcus*, *Streptococcus*, and *Haemophilus* species—pathogens that were absent in unused devices [5]. However, traditional culture techniques are limited by their inability to grow approximately 70% of the known bacterial species [8]. To overcome these limitations, researchers have progressively relied on 16S rRNA gene sequencing. This culture-independent approach targets a gene that is universally present in bacteria, providing a high-resolution map of microbial diversity [9,10]. In line with this, a previous 16S rRNA gene sequencing study on jet nebulizers used by patients with pneumonia or exacerbations of chronic obstructive pulmonary disease (COPD) during hospitalization has identified diverse bacterial communities, including *Stenotrophomonas*, *Rhizobium*, and *Ralstonia*, after only 48 h of use [11].

Despite growing evidence of bacterial contamination, the specific microbial ecology of these inhaler devices remains under-researched. To our knowledge, no study has utilized 16S rRNA gene sequencing to compare the microbial communities of pMDI and Respimat^®^ devices over an extended period. We hypothesized that both devices are susceptible to contamination during use and that the pMDI would exhibit a higher bacterial burden due to its open-valve design. Consequently, this study employed 16S rRNA gene sequencing to characterize and quantify the bacterial profiles of both devices after 30 days of routine use by patients with non-cystic fibrosis bronchiectasis.

## 2. Materials and Methods

### 2.1. Study Design and Participants

This prospective cohort study recruited 17 patients aged 57–77 years who attended the Bronchiectasis Outpatient Clinic of Ulsan University Hospital (Ulsan, Republic of Korea) between October 2018 and March 2019. All included subjects presented with (i) a medical history consistent with bronchiectasis and chronic airway disease requiring inhaler therapy (cough, chronic sputum production, dyspnea, and/or recurrent respiratory tract infections) and (ii) a chest computed tomography (CT) scan demonstrating bronchiectasis in one or more lung lobes [12]. Disease severity was characterized using the FACED score [13], and the radiological extent of bronchiectasis was graded using the modified Reiff score across the six lung lobes [14]. Patients initiating inhaler therapy were trained by a clinical pharmacist on how to properly use either pMDI or Respimat^®^ devices according to a standardized inhaler education checklist (Table S1). All patients provided written informed consent, and the study was approved by the Institutional Review Board and Ethics Committee of Ulsan University Hospital (IRB file no. 2018-10-011-005). Of the enrolled participants, seven were lost to follow-up or did not return their inhalers; thus, 10 subjects provided their inhalers for microbiome analysis. Because two participants used both inhaler types, a total of 12 inhalers (six pMDIs and six Respimat^®^ devices) were retrieved for microbiome analysis after 30 days. In addition, eight unused devices that had never been dispensed to a patient (six pMDIs and two Respimat^®^ devices) were included as baseline controls. The pMDI devices included one Flutiform^®^ (fluticasone/formoterol; Mundipharma, Cambridge, UK), one Alvesco^®^ (ciclesonide; Covis Pharma, Zug, Switzerland), three Symbicort Rapihaler^®^ (budesonide/formoterol; AstraZeneca AB, Södertälje, Sweden), and one Foster^®^ (beclometasone/formoterol; Chiesi Farmaceutici S.p.A., Parma, Italy); all Respimat^®^ devices contained tiotropium (Spiriva^®^). Additionally, to address potential sources of contamination during sample processing, we processed four sterile-filtered phosphate-buffered saline (PBS) samples concurrently as negative controls.

### 2.2. Sample Processing

Participants were instructed to return their inhaler devices after 30 days of routine use, upon which they received a replacement device. Following collection, all inhalers were exposed to ultraviolet (UV) light for a minimum of 1 h (up to overnight) to mitigate potential surface contamination during the subsequent DNA extraction process [15].

To access the internal drug-propellant mixture, the aluminum canisters of the pMDIs were perforated and opened within a Class II biosafety cabinet using sterile, heavy-duty shears. Because of the structural design of the Respimat^®^ device, perforation was not required; the cartridge cap was removed to allow direct access to its liquid contents.

The initial liquid volumes recovered from the pMDI and Respimat^®^ devices reflecting their distinct formulations and internal architectures. Upon collection, the total liquid volume from each device was centrifuged for 20 min at 3500× *g*. The supernatant was discarded, and the resulting pellet was resuspended in phosphate-buffered saline (PBS). DNA extraction was performed using the DNeasy Blood & Tissue Kit (Qiagen, Venlo, The Netherlands) integrated with a mechanical lysis procedure [16]. Mechanical disruption was achieved via bead beating using a Mini-BeadBeater (BioSpec, Bartlesville, OK, USA) for 45 s. The concentration and purity of the extracted DNA were assessed using spectrophotometry (NanoDrop; Thermo Fisher Scientific, Wilmington, DE, USA). Samples with insufficient DNA yield (*n* = 4; two from pMDI and two from Respimat^®^ devices) were further purified and concentrated using the DNA Clean & Concentrator Kit (Zymo Research Corp., Irvine, CA, USA) according to the manufacturer’s instructions. Because qPCR detects target sequences at substantially lower template concentrations than are required for amplicon library preparation, a sample could be quantifiable by qPCR yet fail to yield a library of sufficient quality for sequencing.

Additional details regarding 16S rRNA gene quantification via qPCR are provided in the Online Supplement.

### 2.3. Microbiome Profiling

Sequencing was performed on the Illumina MiSeq platform (2 × 250 bp paired-end reads) at ChunLab, Inc. (Seoul, Republic of Korea) [17]. Raw sequence data were processed using the Quantitative Insights into Microbial Ecology 2 (QIIME 2^®^, v. 2026.1) pipeline [18]. Sequence denoising, quality filtering, and chimera removal were conducted using the Divisive Amplicon Denoising Algorithm 2 (DADA2), with the resulting sequences merged and resolved into amplicon sequence variants (ASVs) [19,20].

To ensure the integrity of the respiratory microbiota analysis, we employed a multi-faceted decontamination strategy. First, ASVs exhibiting less than 80% alignment with the SILVA bacterial database (SSU Ref NR 99, v. 138.2) [21] were excluded to eliminate non-bacterial DNA. To mitigate technical noise and potential PCR artifacts, a prevalence filter was applied, excluding any ASV that was not present in at least two separate samples across the entire sample set (including both inhaler and negative control samples). Potential contaminants were further identified and removed using the *decontam* R package (v. 1.26.0) [20]. A combined identification approach flagged contaminants based on DNA concentration and sequence prevalence across samples, utilizing probability thresholds of 0.1 for both parameters. Subsequently, SourceTracker2 was used to estimate the proportional contribution of bacterial sequences from the oral environment [22]. For SourceTracker2 analyses, publicly available sequencing data from oral wash samples of 25 healthy participants served as the oral source reference (BioProject PRJNA918386) [23].

Taxonomic classification of ASVs was assigned using a naïve Bayes classifier trained on the SILVA database, as described above [24]. The final QIIME 2^®^ outputs, comprising the ASV table and taxonomic annotations, were exported to R (v. 4.5.3) for downstream analysis using the *phyloseq* (v. 1.54.2) and *vegan* (v. 2.7.3) packages [25,26].

Microbial α-diversity was compared between inhaler devices (pMDI vs. Respimat^®^) using four metrics: observed ASV (richness), Shannon index, Pielou’s evenness index, and Faith’s phylogenetic diversity (PD) [27]. The microbial community structure (β-diversity) was analyzed using unweighted Unifrac distances [28]. This distance matrix was selected because it relies on community membership (presence vs. absence) rather than abundance, providing high sensitivity for low-abundance taxa [29]. Principal Coordinate Analysis (PCoA) was performed on this distance matrix to visualize compositional differences between the pMDI and Respimat^®^ microbial communities.

Finally, differentially abundant features between the inhaler groups were investigated using the analysis of compositions of microbiomes with bias correction (ANCOM-BC) [30]. Analysis was performed at the ASV level rather than on a collapsed table at the phylum or genus level to maximize taxonomic resolution. All *p*-values were adjusted using the Benjamini–Hochberg (BH) procedure to control the false discovery rate (FDR) [31]. Taxonomic annotations at the phylum and genus levels were subsequently assigned to all differentially abundant ASVs.

### 2.4. Statistical Analysis

Categorical variables were presented as counts and percentages, and continuous variables were expressed as medians with interquartile ranges (IQRs). Total 16S bacterial loads (copies/mL) were compared across groups (negative control, *n* = 4; Respimat^®^, *n* = 6; pMDI, *n* = 6) using the Kruskal–Wallis test. α-diversity metrics were compared between the pMDI and Respimat^®^ groups using the Mann–Whitney U test. Microbial community structure (β-diversity) was compared between inhaler groups using permutational multivariate analysis of variance (PERMANOVA) via the *adonis2* function in the vegan R package. The PERMANOVA and ANCOM-BC models were adjusted for a history of bronchiectasis exacerbations in the preceding year as a covariate. Additional covariates were not included to prevent model overfitting, given the small sample size.

Statistical significance was set at *p* < 0.05. All microbiome-related plots and statistical visualizations were generated using GraphPad Prism (v. 10.0; GraphPad Software, Inc., Boston, MA, USA) and R (v. 4.5.3).

## 3. Results

### 3.1. Baseline Characteristics of Participants and Study Design

**Figure 1:**
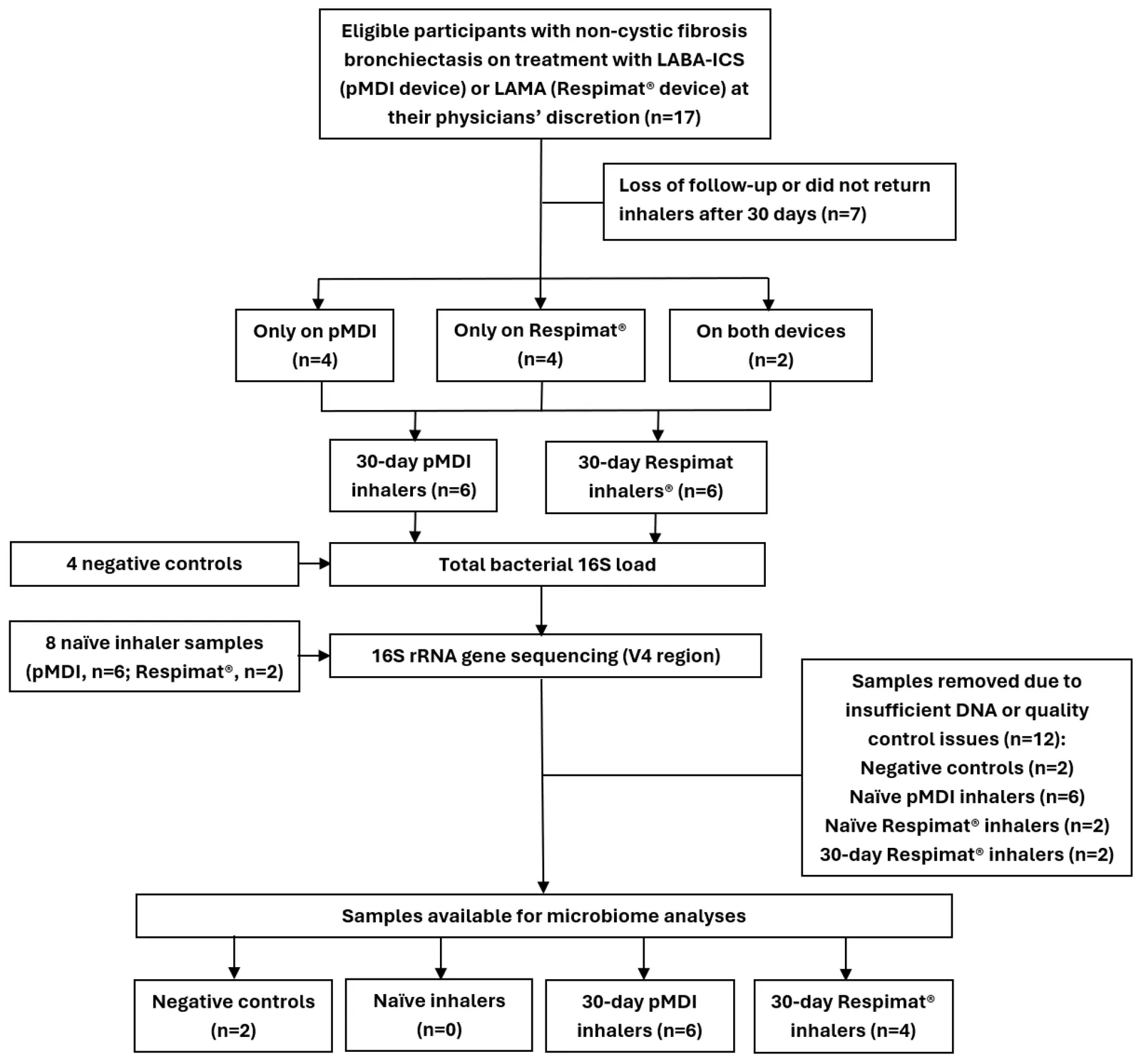
Flowchart of the study participants and inhaler sample collection. The negative control samples consisted of sterile-filtered phosphate-buffered saline. Abbreviations: ICS = inhaled corticosteroids; LABA = long-acting β2-agonist; LAMA = long-acting muscarinic antagonist; pMDI = pressurized metered-dose inhaler.

Of the 17 patients recruited, ten completed the study protocol and returned their devices; because two participants used both device types, this yielded 12 used inhalers (pMDI, *n* = 6; Respimat^®^, *n* = 6). Bacterial DNA was amplifiable by universal-primer qPCR in all 12 used devices, which were therefore all included in the total bacterial-load analysis. Two of the used Respimat^®^ samples, however, yielded DNA insufficient for amplicon library preparation and did not pass sequencing quality control; these, together with all eight unused inhaler samples (pMDI, *n* = 6; Respimat^®^, *n* = 2), were excluded from the sequencing analyses. In total, 10 used inhaler samples (six pMDIs and four Respimat^®^ devices), obtained from eight participants, were included in the final microbiome analyses. The sample distribution and study workflow are illustrated in Figure 1.

Demographic and clinical characteristics are summarized in Table 1. The study population consisted primarily of older adults (median age: 64.5 years) with a marked female predominance (90%). Overall lung function was relatively preserved, with a median pre-bronchodilator FEV_1_ of 71% predicted. Baseline disease severity, assessed using the FACED score (FEV_1_ percentage predicted, age, chronic *P. aeruginosa* airway infection, radiological extension, and dyspnea) and the modified Reiff score for radiological extension, was comparable between the pMDI and Respimat^®^ groups. Furthermore, the prevalence of chronic airway infection with *P. aeruginosa* did not differ significantly between the groups, ensuring a balanced clinical baseline for microbiome analyses.

### 3.2. Microbial Diversity Analyses

A total of 683,886 raw reads were imported into the QIIME 2^®^. Following sequence quality control with the DADA2 algorithm, 515,996 reads (848 ASVs) were retained, including two MOCK samples and two negative controls. During this process, five ASVs (19 reads) were excluded because of insufficient alignment (<80% identity) to the SILVA bacterial database.

After removing the MOCK samples and excluding ASVs detected in only a single sample, 244 ASVs (363,589 reads) were identified. An additional 10 ASVs (1080 reads) were identified as potential contaminants via the *decontam* R package and were subsequently removed (Table S2). Following the exclusion of negative control samples (*n* = 2) and ASVs lacking phylum-level annotation, 303,632 reads (231 ASVs) across 10 inhaler samples were retained for the final analyses. The minimum and median read counts per inhaler sample were 16,720 and 54,344, respectively.

**Figure 2:**
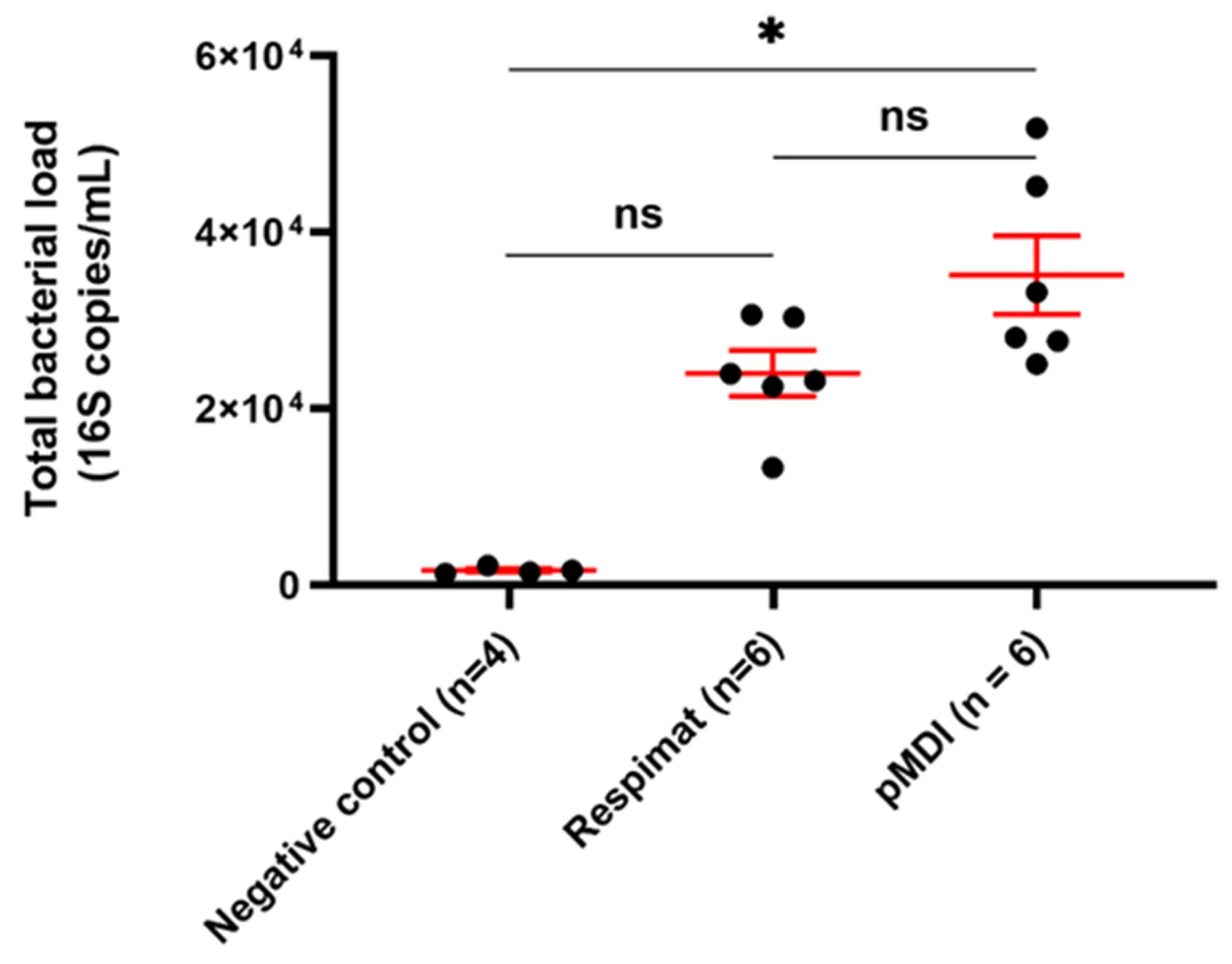
Total 16S rRNA gene copy numbers in negative controls and in used inhaler devices after 30 days of use. Bacterial load was quantified by qPCR using universal bacterial 16S rRNA gene primers (Online Supplement), and all 12 used inhalers retrieved from the 10 participants who completed the protocol were included (negative controls, *n* = 4; Respimat^®^, *n* = 6; pMDI, *n* = 6). Bacterial DNA was quantifiable in every used device, including the two Respimat^®^ samples that subsequently yielded DNA insufficient for library preparation and were therefore excluded from the sequencing analyses. The red bars represent the median bacterial load in each group. The overall *p*-value (<0.001) was obtained using the Kruskal–Wallis test. Post-hoc multiple pairwise comparisons were performed using Dunn’s test to obtain adjusted *p*-values (ns: *p* ≥ 0.05, * *p* < 0.05).

Total bacterial load, quantified by qPCR in all 12 used inhalers (pMDI, *n* = 6; Respimat^®^, *n* = 6) and in the negative controls (*n* = 4), varied significantly across groups (*p* < 0.001; Figure 2). Pairwise comparisons revealed that bacterial loads in 30-day Respimat^®^ devices remained comparable to those in the negative controls (adjusted *p* = 0.15). In contrast, the 30-day pMDI samples exhibited a significantly higher bacterial burden than the negative controls (adjusted *p* = 0.003). Despite this increase, inter-device analysis showed no significant difference in 16S bacterial loads between pMDI and Respimat^®^ samples after 30 days of use (adjusted *p* = 0.43).

**Figure 3:**
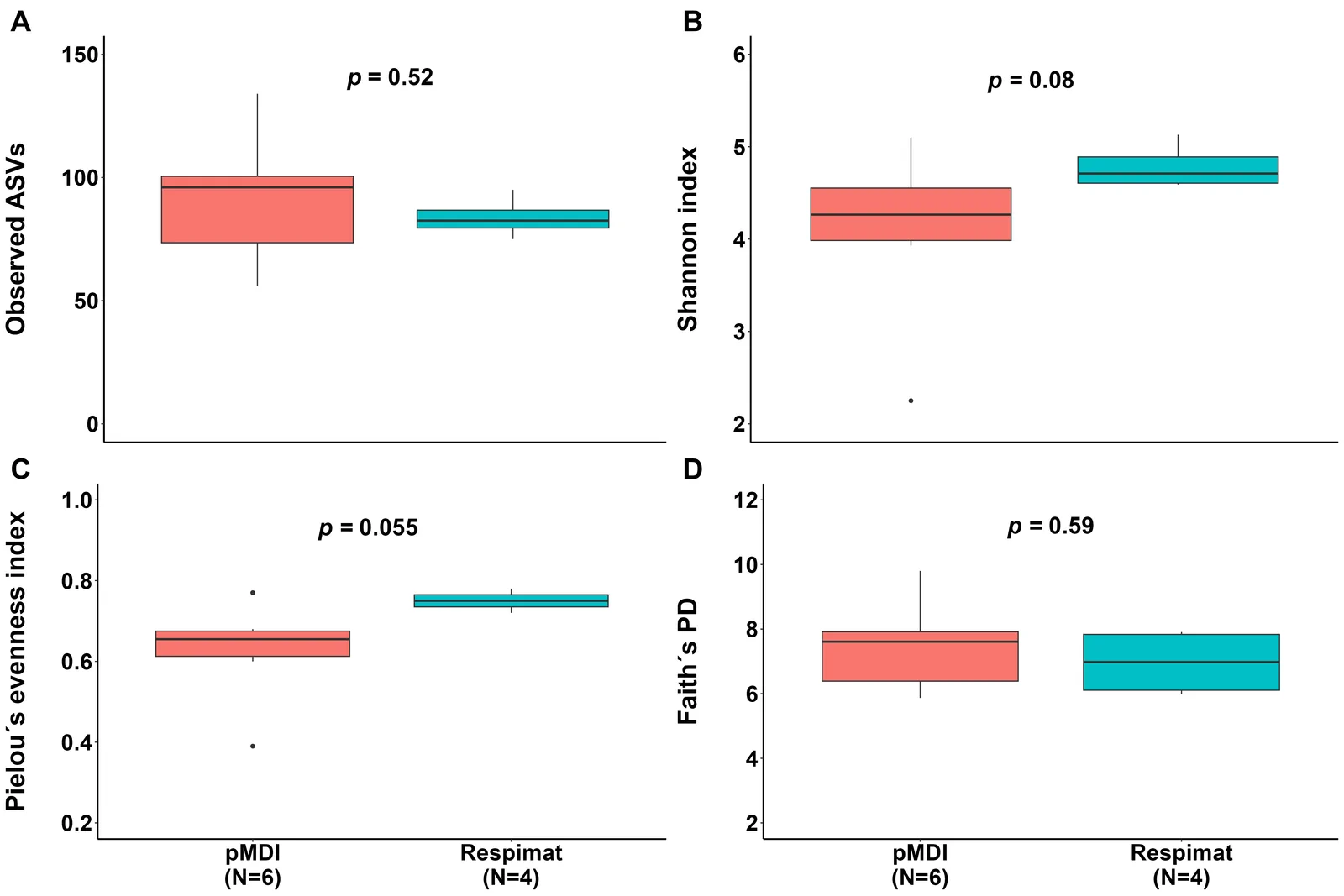
Comparison of α-diversity metrics between pMDI and Respimat inhaler groups. Box and whisker plots show the median (horizontal line) and interquartile range (IQR) with whiskers extending to 1.5 x IQR; dots represent values outside that range. In all panels, red boxes denote pMDI devices (*n *= 6) and blue boxes denote Respimat® devices (*n*= 4). (**A**) observed amplicon sequence variants (ASVs), (**B**) Shannon diversity index, (**C**) Pielou’s evenness index, and (**D**) Faith’s phylogenetic diversity (Faith’s PD). All *p*-values shown were obtained using the Mann–Whitney U test. Abbreviations: ASVs = amplicon sequence variants; Faith's PD = Faith’s phylogenetic diversity; IQR = interquartile range; pMDI = pressurized metered-dose inhaler.

**Figure 4:**
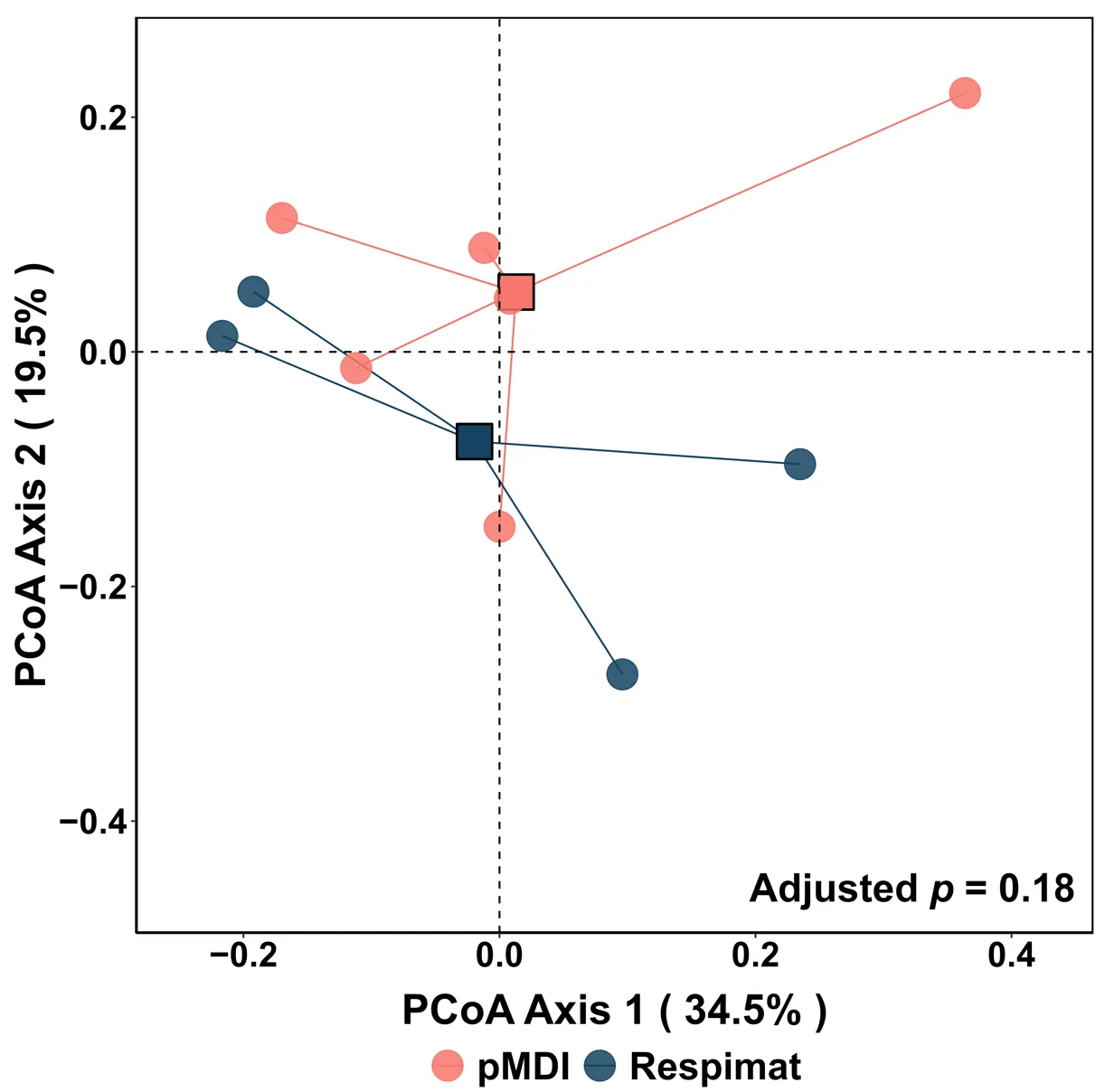
Comparison of β-diversity (microbial communities) based on unweighted UniFrac distances between inhaler groups. Circles represent individual samples and squares represent the group centroids, that is, the average position of a group of samples within an ordination plot, calculated from their multivariate dissimilarity; red denotes pMDI devices (*n* = 6) and blue denotes Respimat® devices (*n* = 4). The *p*-value was obtained using a permutational multivariate ANOVA (PERMANOVA; via the *adonis2* function from the Vegan R package [25]) test with 1000 permutations. The PERMANOVA model was adjusted for bronchiectasis exacerbations in the preceding year. Abbreviations: PCoA = Principal Coordinate Analysis.

Regarding alpha-diversity, a non-significant trend toward a reduced Shannon index and Pielou’s evenness index was noted in pMDI samples compared to Respimat^®^ devices (*p* = 0.08 and *p* = 0.055, respectively). In contrast, no significant differences were detected in microbial richness or Faith’s phylogenetic diversity (PD) between the inhaler groups (Figure 3). Beta-diversity analysis indicated that the microbial communities did not significantly differ between the two inhaler devices (adjusted *p* = 0.18) (Figure 4).

**Figure 5:**
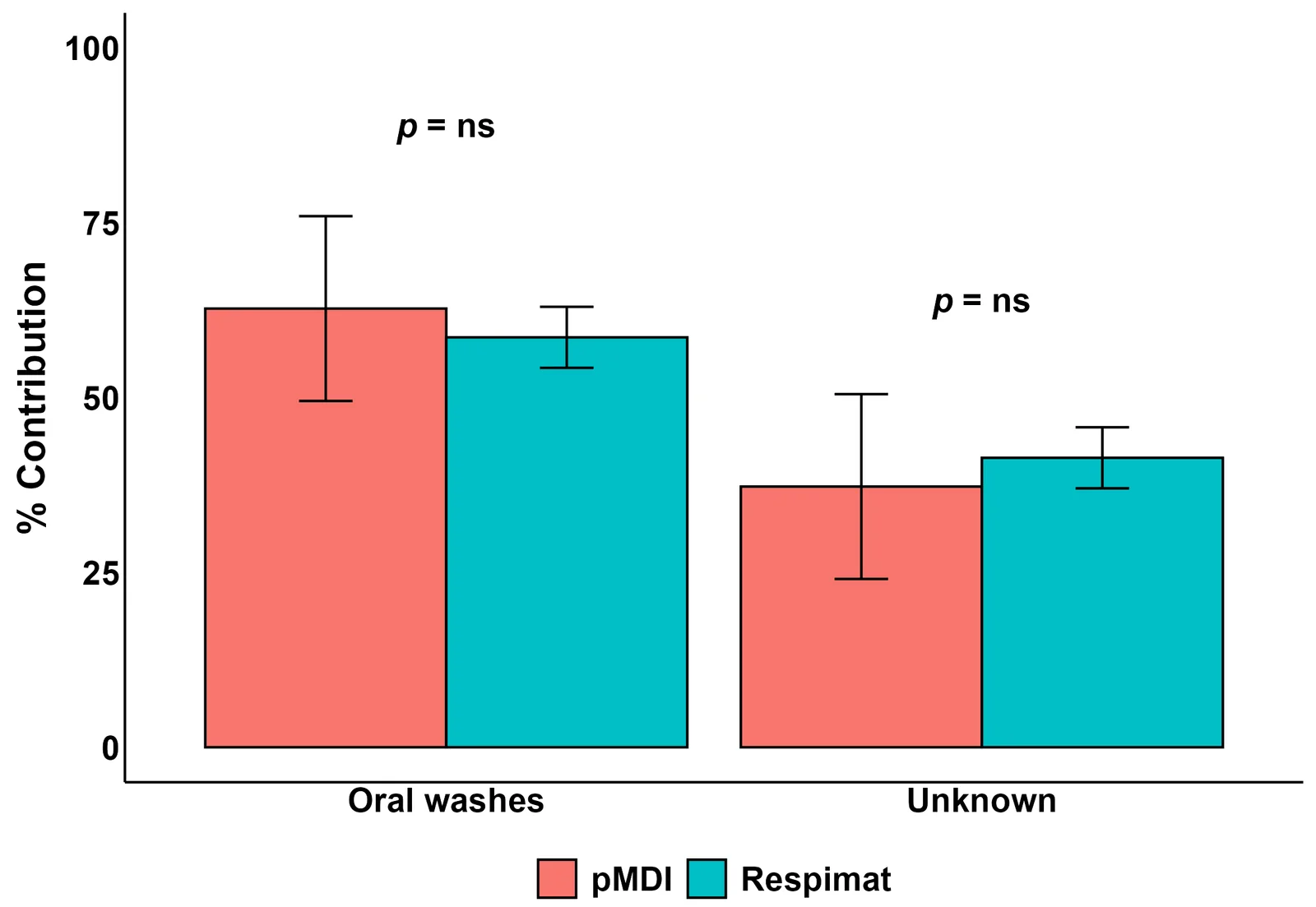
Percentage contribution of microbial profiles from oral and unknown sources to inhaler samples after 30 days of use. The oral microbiome data used in these analyses were retrieved from publicly available sequencing data of oral wash samples collected from 25 healthy participants (BioProject PRJNA918386) [23]. Error bars represent the standard error of the mean (SEM). All *p*-values were obtained using the Mann–Whitney U test. Abbreviations: ns = non-significant (*p* ≥ 0.05).

The SourceTracker2^®^ tool was used to estimate the proportional contribution of the oral microbiota (derived from oral washes) to the microbial communities recovered from the 30-day inhaler samples (Figure 5). The estimated oral contribution was numerically higher in pMDI samples than in Respimat^®^ samples (mean ± SEM: 62.7 ± 13.2 vs. 58.6 ± 4.4%, respectively), although this difference did not reach statistical significance (*p* = 0.46). Notably, these results indicate that oral microbial acquisition accounted for approximately 60% of the microbial profiles detected in both pMDI and Respimat^®^ devices after 30 days of use.

### 3.3. Relative Abundance Taxa Comparisons

**Figure 6:**
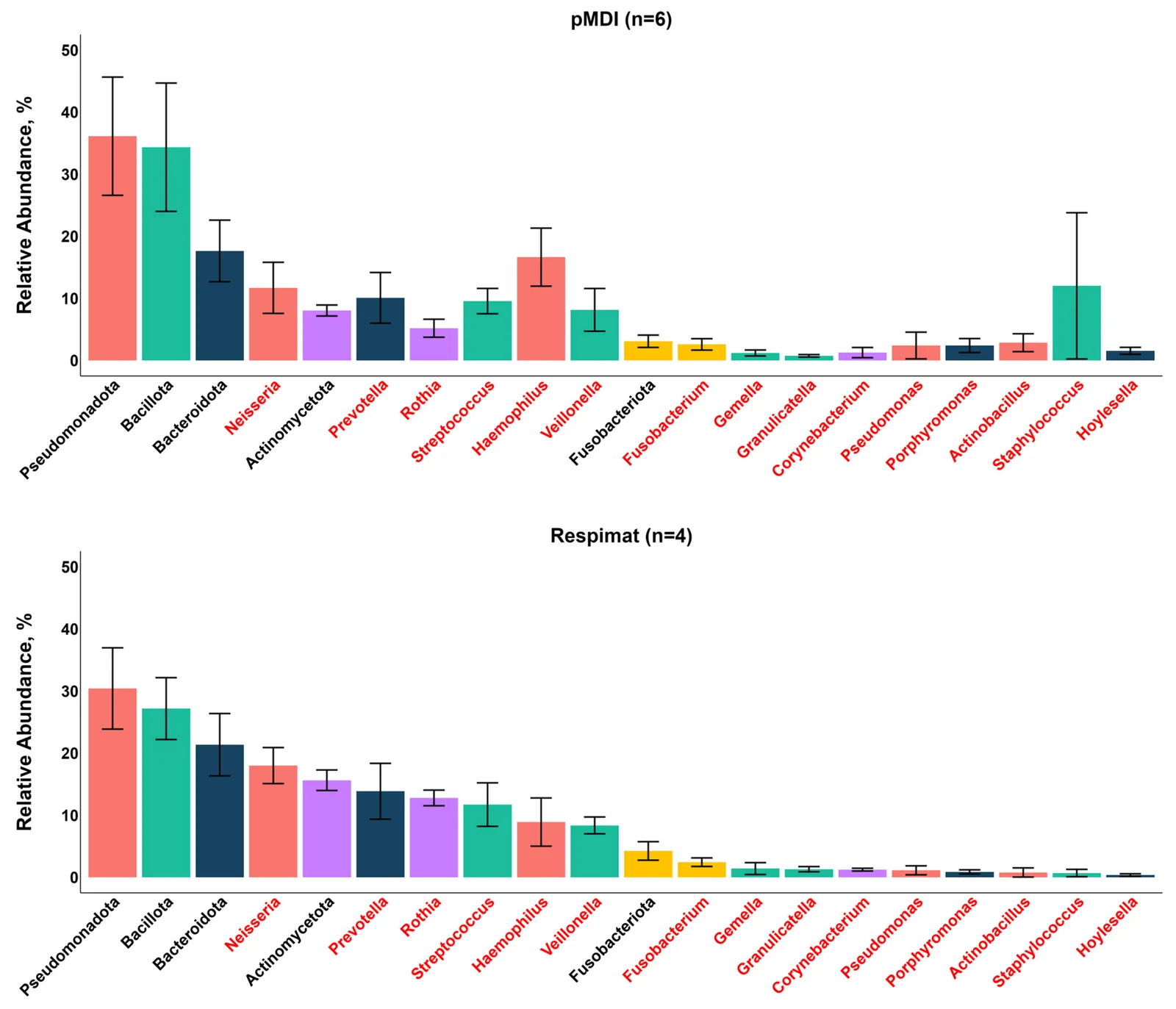
Relative abundance of the most prevalent phyla and genera in pMDI (upper panel) and Respimat^®^ (lower panel) samples following 30 days of use. Data are presented as mean percentages of total sequencing reads, with error bars representing the standard error of the mean (SEM). No statistically significant differences in relative abundance were observed between the device groups for the dominant taxa, although a marginal trend toward increased *Actinomycetota* abundance occurred in the Respimat^®^ group (adjusted *p* = 0.053). All *p*-values were determined using the Mann–Whitney U test. Note: Taxonomic classifications for the phyla *Proteobacteria* and *Firmicutes* were updated to *Pseudomonadota* and *Bacillota*, respectively, in accordance with the latest SILVA bacterial database (release 138.2, SSU Ref NR 99) [18]. Taxon labels are shown in black for phyla and in red for genera.

Analysis of the 10 inhaler samples revealed that the most prevalent phyla by mean relative abundance were *Pseudomonadota* (35.4%), *Bacillota* (29.7%), *Bacteroidota* (19.9%), *Actinomycetota* (10.7%), and *Fusobacteriota* (3.5%) (Table S3). At the genus level, the bacterial community was dominated by *Haemophilus* (15.2%), *Neisseria* (14.0%), *Prevotella* (12.0%), *Streptococcus* (10.7%), and *Veillonella* (9.7%), which collectively accounted for more than 60% of the total sequencing reads (Table S4). The distribution of these dominant taxa across the inhaler groups is shown in Figure 6 (detailed in Tables S5 and S6). Despite a marginal trend toward increased *Actinomycetota* abundance in Respimat^®^ samples (adjusted *p* = 0.053), no statistically significant differences in taxonomic composition were observed between the inhaler groups at either the phylum or genus level. Regarding *Pseudomonas*, sequences were detected in 5/6 pMDI samples (83.3%) compared to 2 of 4 Respimat^®^ samples (50.0%) (*p* = 0.50, Fisher’s exact test). Notably, the pMDI user with documented chronic *P. aeruginosa* airway infection (via sputum culture) yielded inhaler samples positive for *Pseudomonas* sequences. However, sequences belonging to the *Pseudomonas* genus were also identified in four additional pMDI inhaler samples from participants with no prior history of chronic airway infection by *P. aeruginosa*. Conversely, of the two Respimat^®^ samples positive for *Pseudomonas*, only one corresponded to a patient with a history of chronic airway infection by *P. aeruginosa* on sputum culture.

As noted above, two participants (8612074 and 1204284) used both pMDI and Respimat^®^ devices, which were retrieved for analysis after 30 days of use. The microbial composition of the paired inhaler samples at the phylum and genus levels is shown in Figure S1. Notably, within each participant, the proportion of reads assigned to each prespecified potentially pathogenic genus was compared to the proportion of all remaining reads between paired pMDI and Respimat^®^ samples. Significant differences in genus-specific read proportions were observed for both participants, as summarized in Table S7.

In participant 8612074, the pMDI sample showed higher relative abundances of *Haemophilus* (33.68% vs. 19.49%; adjusted *p* < 0.001) and *Pseudomonas* (13.15% vs. 1.51%; adjusted *p* < 0.001) compared to the Respimat^®^ sample. Conversely, the Respimat^®^ sample showed higher relative abundances of *Streptococcus* (9.12% vs. 5.58%; adjusted *p* < 0.001) and *Staphylococcus* (0.33% vs. 0.16%; adjusted *p* < 0.001).

A similarly marked divergence in genus-level composition was observed in participant 1204284. The pMDI sample was dominated by *Staphylococcus*, with a relative abundance of 70.90% compared to 2.45% in the Respimat^®^ sample (adjusted *p* < 0.001). In contrast, the Respimat^®^ sample showed higher relative abundances of *Haemophilus* (9.91% vs. 1.06%; adjusted *p* < 0.001), *Streptococcus* (20.73% vs. 3.83%; adjusted *p* < 0.001), and *Pseudomonas* (3.02% vs. 0.51%; adjusted *p* < 0.001) compared to the corresponding pMDI sample.

### 3.4. Differential Taxa Abundance Analyses

**Figure 7:**
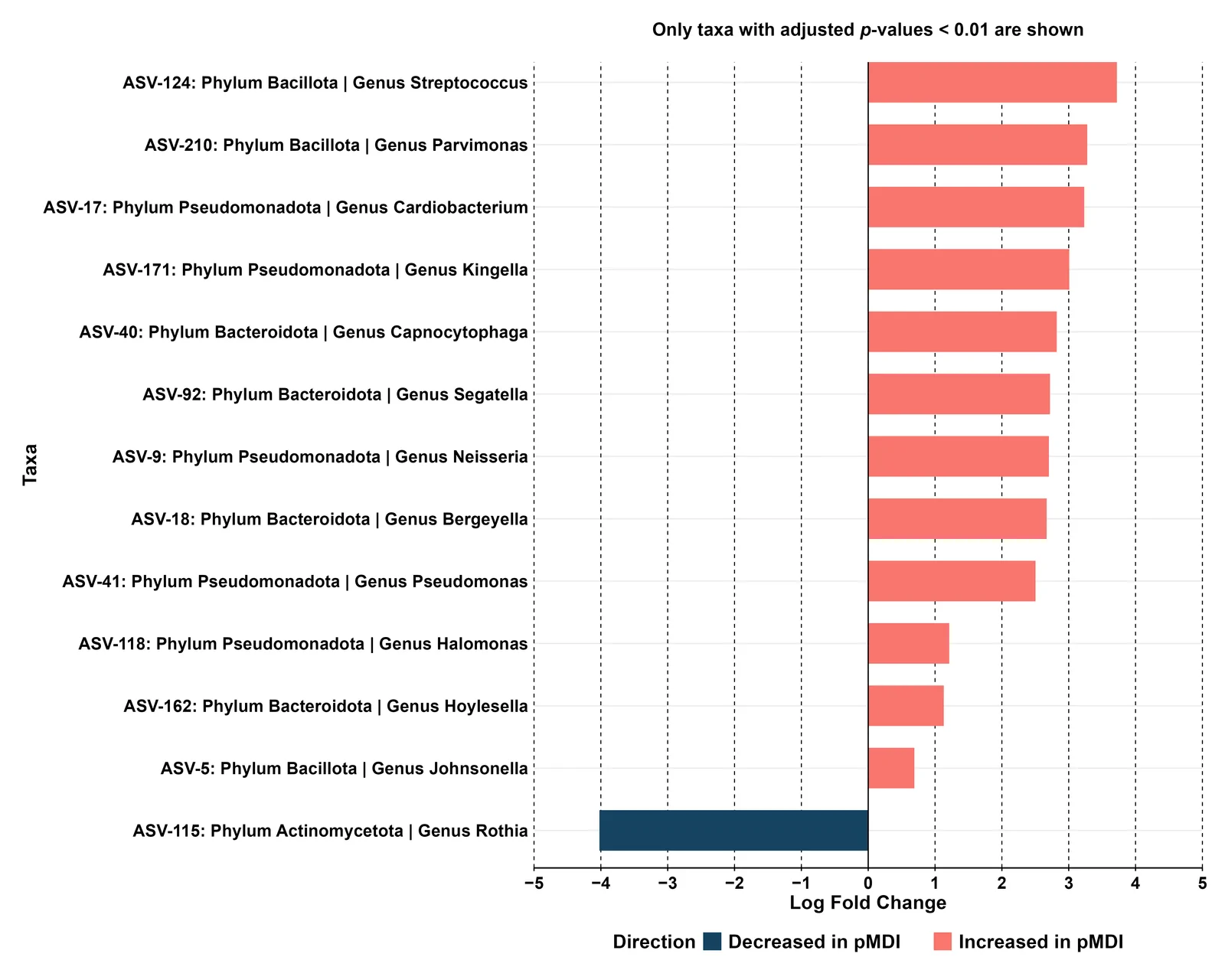
Differential microbiome analysis using ANCOM-BC at the ASV level comparing pMDI (*n* = 6) and Respimat^®^ (*n* = 4) devices after 30 days of use. The ANCOM-BC model was adjusted for bronchiectasis exacerbations in the preceding year.

Differential abundance analysis using the ANCOM-BC pipeline identified distinct microbial signatures characterizing each inhaler type (adjusted *p* < 0.01; Figure 7). Several ASVs were significantly enriched in the pMDI samples compared to those in the Respimat^®^ samples, most notably those belonging to the genera *Parvimonas* (ASV-210) and *Streptococcus* (ASV-124). Furthermore, the pMDI microbial profile was predominantly defined by a robust enrichment of the *Pseudomonadota* phylum, which accounted for the highest number of significantly over-represented taxa, including ASV-17 (*Cardiobacterium*), ASV-9 (*Neisseria*), and ASV-41 (*Pseudomonas*). Conversely, Respimat^®^ devices were characterized by a significant enrichment of ASV-115 (*Rothia*). These findings indicate that while both devices facilitate oral microbial acquisition, the pMDI environment preferentially favors a broader consortium of obligate anaerobes—such as *Parvimonas*—alongside potentially pathogenic bacteria, including members of the *Pseudomonas* genus.

## 4. Discussion

Previous studies have reported the presence and quantity of bacteria colonizing inhaler devices. However, these investigations relied exclusively on culture-dependent methods to sample the mouthpiece [5,6,7,32] or canister spray tip [7,32] of pMDI devices. Furthermore, the pMDIs analyzed in these studies were retrieved from patients with various respiratory conditions, and the specific duration of device use prior to analysis was not clearly defined. To the best of our knowledge, only one study has utilized 16S rRNA sequencing to investigate inhaler microbiome profiles; however, that study analyzed only jet nebulizers in patients hospitalized for pneumonia or COPD exacerbations [11]. Additionally, in that investigation, sampling of the jet nebulizer components was performed after only 48 h of use, which likely underestimates the microbial colonization occurring in real-world clinical practice. Here, we extend these previous observations by investigating the microbial profiles of two distinct devices (pMDI and Respimat^®^) using 16S rRNA sequencing of the liquid contents after 30 days of use by patients with non-cystic fibrosis bronchiectasis.

Our primary findings demonstrated that although both inhaler types acquired human-derived bacteria over 30 days of clinical use, only the pMDI exhibited a significantly higher bacterial burden than the ultrapurified water of the negative controls (adjusted *p* < 0.05). In contrast, although bacterial loads in Respimat^®^ devices also increased numerically over the 30-day period, this increase did not reach statistical significance relative to negative controls (adjusted *p* = 0.15). Rather than indicating an absence of contamination, these data suggest that Respimat^®^—likely owing to its distinct structural design—may be relatively more resistant to microbial accumulation than pMDIs during one month of active clinical use. A further observation is consistent with this interpretation. Amplicon library preparation failed for two of the six used Respimat^®^ devices but for none of the six pMDIs, even though bacterial DNA was quantifiable by qPCR in all 12 devices. Because library preparation requires substantially more template than qPCR, this asymmetry most parsimoniously reflects lower bacterial DNA content in the Respimat^®^ samples rather than extraction failure or PCR inhibition. We note, however, that library-preparation failure can also reflect DNA quality or extraction efficiency, and we regard this observation as supportive rather than confirmatory.

Furthermore, SourceTracker2 analysis attributed approximately 60% of the recovered microbial sequences to the oral cavity, indicating that both inhaler devices can act as substantial reservoirs for oropharyngeal flora. Notably, these findings persisted despite standardized instructions provided to all participants regarding stringent daily device hygiene.

In our study, unused control inhalers (*n* = 8; pMDI = 6, Respimat^®^ = 2) yielded DNA concentrations below the limit of detection for microbial profiling; consequently, these samples were excluded from the downstream analyses. Our findings align with those of Borovina et al. [5] and Swanson et al. [11], who also reported negligible microbial presence in naïve respiratory devices. Specifically, Borovina et al. found no microbial growth after inoculating swabs from the external and internal surfaces of unused devices with selective growth media. Similarly, Swanson et al. applied 16S rRNA sequencing to sterile swabs from jet nebulizers and found them to be essentially sterile at baseline. Taken together, these observations validate our stringent extraction and processing protocols, confirming that the microbial signatures identified in the used devices were not the result of environmental or procedural contamination.

Microbial communities between pMDI and Respimat^®^ inhaler samples did not significantly differ on day 30 (Figure 4) (adjusted *p* = 0.18). However, alpha diversity analyses provided further insights into device-specific microbial ecology. Both the Shannon diversity index and Pielou’s evenness index exhibited a trend toward lower α-diversity in pMDI samples than in Respimat^®^ alternatives (*p* = 0.08 and *p* = 0.055, respectively). Given that reduced α-diversity is a recognized hallmark of microbial dysbiosis, frequently associated with disease progression and poor clinical outcomes [23,33,34], we suggest that pMDI devices may be more susceptible to microbial simplification and the subsequent dominance of specific taxa. These observations should be regarded as exploratory and hypothesis-generating rather than evidence of a device-specific effect on microbial diversity. Larger studies with adequate statistical power are required to determine whether differences in α-diversity between inhaler types are reproducible and clinically relevant.

This selective pressure is also supported by differential abundance analyses carried out using the ANCOM-BC pipeline, which identified 12 distinct ASVs significantly enriched (adjusted *p* < 0.01) in pMDIs compared to only one in Respimat^®^ devices (Figure 7). The pMDI signature was characterized by a robust overrepresentation of obligate anaerobes typically associated with oral biofilms and periodontal disease, such as *Parvimonas* (ASV-210) [35] and *Cardiobacterium* (ASV-17) [36]. These findings are clinically relevant, as a growing body of evidence links oral dysbiosis with respiratory disease progression, potentially through microaspiration of biofilm-associated bacteria into the lower airways [37]. Moreover, ANCOM-BC identified an enrichment of potentially pathogenic taxa in pMDI samples. Identification via the NCBI Basic Local Alignment Search Tool (BLAST) [38], available at https://blast.ncbi.nlm.nih.gov/Blast.cgi (accessed on 2 August 2026), confirmed ASV-124 as *Streptococcus constellatus*, a common oral inhabitant, and, more critically, ASV-41 as *Pseudomonas aeruginosa*. The presence of the latter is of significant prognostic importance, as chronic *P. aeruginosa* infection is associated with accelerated lung function decline, increased exacerbation frequency, and higher mortality in patients with non-cystic fibrosis bronchiectasis [12,39,40].

Finally, the intra-subject paired analyses provide further evidence of differential bacterial acquisition between devices during use (Figure S1; Table S7). For instance, in participant 1204284, the pMDI microbial profile was nearly monopolized by the genus *Staphylococcus* (70.9% relative abundance); conversely, the same participant’s Respimat^®^ device yielded a negligible 2.5% *Staphylococcus* abundance, in contrast to a higher relative abundance of *Pseudomonas* and *Streptococcus*.

These results demonstrate that although the microbial source (the patient’s oral cavity or environment) is identical, both inhaler devices, despite undergoing similar cleaning procedures, may be associated with over- or underrepresentation of different bacterial ASVs. However, the mechanistic basis for these device-specific differences remains unclear.

Because any microbial ingress into the sealed Respimat^®^ cartridge must occur retrograde through its fine uniblock microchannels (on the order of ~5 × 8 µm) [1], a size-selective effect favoring smaller single cells over larger oral aggregates or cluster-associated particles is mechanically plausible. However, our 16S data and microbiome analyses do not indicate that these differences are driven solely by bacterial morphology; rather, the overall pattern of higher bacterial loads and more pMDI-enriched ASVs is consistent with potential device-specific selective entry and retention of oral microbes. This interpretation should be regarded as hypothesis-generating, as our 16S-based approach could not distinguish the respective contributions of microbial size or shape, aggregation state, viability, or other device-specific factors, such as internal fluid dynamics and valve design.

Our study had several limitations. The sample size was relatively small; we analyzed a total of 10 used inhalers (six pMDI and four Respimat^®^) retrieved from eight participants who completed the 30-day protocol. However, this is the first study to utilize culture-independent 16S rRNA sequencing to evaluate handheld maintenance inhalers in a clinical population of adult patients with bronchiectasis over a standardized period. Furthermore, baseline microbial profiles were unavailable for unused inhalers, thus precluding us from investigating any potential microbial shifts over usage from naïve inhalers. Nevertheless, the absence of a 16S signal in unused samples strongly suggests they were initially sterile (as expected and previously reported [5,11]), and the detected microbial communities were acquired during patient use. Moreover, it is important to point out that 16S rRNA amplicon sequencing detects bacterial DNA regardless of cellular viability. Thus, the identification of opportunistic taxa, such as *Pseudomonas aeruginosa*, indicates molecular presence rather than viable colonization or active infection. Distinguishing viable, metabolically active bacteria from non-viable DNA remnants requires culture-based methods or viability-PCR assays. Consequently, classifying these handheld inhalers as reservoirs for viable pathogens remains speculative based on sequencing data alone. Another confounding factor is that pMDIs delivered different ICS-based formulations, whereas all Respimat^®^ devices contained tiotropium. Because device type and drug formulation varied together, their effects on the observed microbial profiles could not be separated. The differences observed between these devices therefore cannot be attributed to device design or aerosolization mechanics alone; all device-specific findings regarding potential microbial acquisition during the 30-day use period should therefore be interpreted with caution. Additionally, we did not directly collect oral washes from our subjects. Consequently, for our SourceTracker2 analyses, we used sequencing data from the oral washes of healthy individuals, which may not perfectly represent the oral microbiome of our specific patient cohort. Moreover, our participants were primarily classified as having mild bronchiectasis according to the FACED score, and only two of the eight participants were identified as having chronic airway infection by *P. aeruginosa*. Therefore, any extrapolation of these findings to patients with more severe disease should be performed with caution. Participant compliance with the prescribed inhalation regimen also could not be objectively determined. Although all participants were instructed to perform daily device hygiene, we could not verify adherence to specific cleaning procedures, nor did we perform a standardized assessment of device cleanliness, in contrast to some previous studies [5,6]. This, however, reflects the real-world nature of our data, and all participants received identical pharmacist-delivered inhaler technique education.

Despite these limitations, the broader clinical implications of our findings warrant consideration. Determining if the choice of inhaler device represents a modifiable risk factor for managing chronic airway diseases remains beyond the purview of this study. Given that pathogenic contamination of domiciliary nebulizers has been previously linked to a nearly twofold increase in the annual rate of COPD exacerbations [6], further large-scale prospective studies are needed. Such research is essential to determine whether and how device-specific microbial profiles directly impact long-term clinical outcomes and exacerbation frequencies in patients with respiratory diseases.

## 5. Conclusions

Following 30 days of clinical use, both inhaler devices showed evidence of harboring diverse microbial communities in their liquid contents. Higher bacterial loads, a statistical trend supporting specific lower α-diversity metrics, and more pronounced alterations in microbial abundance of several ASVs in pMDI devices compared to Respimat^®^ devices suggest that the former may be more susceptible to bacterial acquisition during use in patients with non-cystic fibrosis bronchiectasis. These findings underscore the need for further prospective investigation to determine whether device contamination influences airway colonization or clinical outcomes in this population.

## Figures and Tables

**Table 1:** Baseline characteristics of study participants.

	Study Population ^a^ (10 Participants)	Respimat^®^ Group ^b^ (5 Participants)	pMDI Group ^c^ (5 Participants)	*p*-Value
Age, years (range)	64.5 (57–77)	67 (63–77)	63 (57–69)	0.11
Female sex, n (%)	9 (90.0%)	4 (80.0%)	5 (100.0%)	1.00
BMI, kg/m^2^ (range)	21.7 (19.8–28.9)	22.5 (19.8–24)	20.8 (19.9–28.9)	0.80
Smoking status				
Non-smokers, n (%)	9 (90.0%)	4 (80.0%)	5 (100.0%)	1.00
Former smokers, n (%)	1 (10.0%)	1 (20.0%)	0 (0.0%)	1.00
Lung function ^d^				
FVC, % of predicted (range)	77.5 (62–98)	72 (62–98)	83 (62–93)	0.83
FEV_1_, % of predicted (range)	71 (48–108)	62 (48–108)	80 (48–91)	0.98
FEV_1_/FVC % (range)	66.5 (52–86)	65 (52–80)	68 (52–86)	0.66
Etiology of bronchiectasis				1.00
Idiopathic	4 (40.0%)	2 (40.0%)	2 (40.0%)	
Post-infectious	5 (50.0%)	3 (60.0%)	2 (40.0%)	
NTM	1 (10.0%)	0 (0.0%)	1 (20.0%)	
Modified Reiff score	7 (3–10)	8 (3–10)	6 (4–10)	0.89
Exacerbation in the previous year	3 (30.0%)	1 (20.0%)	2 (40.0%)	1.00
FACED score	1.5 (0–3)	1.5 (1–3)	1 (0–3)	0.57
*Pseudomonas aeruginosa* chronic airway infection	2 (20.0%)	1 (20.0%)	1 (20.0%)	1.00

Data are presented as number (%) or median (range: minimum, maximum). ^a^ Ten participants provided inhalers after 30 days of use; however, during sequence quality filtering of 16S rRNA gene sequencing data, inhaler samples from two participants were excluded from downstream microbiome analyses. Thus, all analyzed inhaler samples were obtained from eight study participants. Moreover, two subjects used both Respimat^®^ and pMDI devices. ^b^ All Respimat^®^ devices were Spiriva^®^ (tiotropium). ^c^ Among subjects using pMDIs, devices included one Flutiform^®^, one Alvesco^®^, three Symbicort Rapihaler^®^, and one Foster^®^. ^d^ Pre-bronchodilator lung function values. Abbreviations: BMI = body mass index; FACED [13] = a seven-point score based on five dichotomized variables, consisting of: F = ${\mathrm{FEV}}_{1}$ % predicted (≤50%, 2 points); A = age (≥70 years, 2 points); C = chronic airway colonization by *P. aeruginosa* (1 point); E = radiological extension (≥2 affected lung lobes, 1 point); and D = dyspnea (≥grade 2 on the Medical Research Council scale, 1 point); ${\mathrm{FEV}}_{1}$ = forced expiratory volume in 1 s; FVC = forced vital capacity; NTM = nontuberculous mycobacteria; pMDI = pressurized metered-dose inhaler.

## Data Availability

All data generated in this study are available from the corresponding author upon request.

## Supplementary Materials

The following supporting information can be downloaded at: https://www.mdpi.com/article/10.3390/biomedicines14092132/s1. References [41,42,43] are cited in the supplementary materials. Table S1. Standardized inhaler technique checklist for pressurized metered-dose inhalers (pMDIs) and Respimat® device. Table S2. Taxonomy annotations at the phylum and genus levels for the 10 amplicon sequence variants (ASVs) identified as potential contaminants using the decontam R package. Table S3. Distribution of the most frequent bacterial phyla across inhaler samples (pooled pMDI and Respimat® devices, *n* = 10). Table S4. Distribution of the most frequent bacterial genera across inhaler samples (considering pooled pMDI and Respimat® devices, *n* = 10). Table S5. Relative abundance of predominant bacterial phyla stratified by inhaler device (pMDI vs. Respimat®). Table S6 Relative abundance of predominant bacterial genera stratified by inhaler device (pMDI vs. Respimat®). Table S7. Within-patient comparison of selected bacterial genera between pMDI and Respimat samples. Figure S1. Relative abundance of dominant bacterial phyla and genera in paired pMDI and Respimat® inhaler samples used by the same participants (*n* = 2) over a 30-day period.
