# Supplementary material for: Inhaler Devices as Potential Bacterial Reservoirs: A 30-Day Microbiota Analysis in Non-Cystic Fibrosis Bronchiectasis
Source: Biomedicines. 2026 Sep 21;14(9):2132. doi: 10.3390/biomedicines14092132 (PMC13604617; doi:10.3390/biomedicines14092132)
Supplement: Supplementary file 1 [file biomedicines-14-02132-s001.zip › biomedicines-4519145-supplementary.pdf]

Online Supplement

Inhaler Devices as Potential Bacterial Reservoirs: A 30-Day Microbiota Analysis in Non-Cystic Fibrosis Bronchiectasis

Jiyeon Baek [1*], Ok Joo Sul [2*], Hye Won Choi [2], Chaeyeong Jung [3], Sunju Lee [4,5], Jinkyeong Park [6], Harim Koo [7], Byungsuk Kwon [7,8], F. S. Leitao Filho [9,10#] and Seung Won Ra [7,11#]

[1] Medical Science, Asan Medical Institute of Convergence Science and Technology, Asan Medical Center, University of Ulsan College of Medicine, Seoul, Republic of Korea

[2] Biomedical Research Center, Ulsan University Hospital, School of Medicine, University of Ulsan College of Medicine, Ulsan, Republic of Korea

[3] Department of Medicine, Soonchunhyang University College of Medicine, Cheonan, Republic of Korea

[4] School of Medical Science, University of Ulsan, Ulsan, Republic of Korea

[5] Biomedical Research Institute, Ulsan University Hospital, Ulsan, Republic of Korea

[6] Department of Pulmonary, Allergy and Critical Care Medicine, Kyung Hee University Hospital at Gangdong, Seoul, Republic of Korea

[7] Department of Medical Science Convergence, Graduate School of Medical Science, Basic-Clinical Convergence Research Institute, University of Ulsan, Ulsan, Republic of Korea

[8] School of Biological Sciences, University of Ulsan, Ulsan, Republic of Korea

[9] Division of Pulmonology, Department of Internal Medicine, Universidade Federal de São Paulo, São Paulo, Brazil

[10] Evidence-Based Medicine and Urgent Medicine Unit, Department of Internal Medicine, Universidade Federal de São Paulo, São Paulo, Brazil

[11] Division of Pulmonology, Department of Internal Medicine, Ulsan University Hospital, University of Ulsan College of Medicine, Ulsan, Republic of Korea

***** These authors (J. Baek and O. J. Sul) contributed equally to this work.

Correspondence: S. W. Ra (docra@uuh.ulsan.kr); F. S. Leitao Filho (fernando.studart@unifesp.br).

1) Absolute quantification analysis: standard curve method for absolute quantification

All 12 used inhalers were included in this analysis. The total bacterial load was quantified by targeting the 16S rRNA gene via qPCR, as previously described [1]. To establish a standard curve, *Escherichia coli* was cultured in Luria-Bertani (LB) medium for 16 h (37°C and 200 rpm) and adjusted to a concentration of 2 x 10^9 colony-forming units (CFU)/mL prior to genomic DNA extraction. Serial 10-fold and 2-fold dilutions of the extracted DNA were prepared, yielding a standard range of 1 x 10^3 to 2 x 10^7 gene copies/μL. Sample DNA was amplified using the universal bacterial 16S rRNA gene primers Total-F (5′-GTGCCAGCMGCCGCGGTAA-3′) and Total-R (5′-GACTACCAGGGTATCTAAT-3′) [2], The resulting cycle threshold (C_t) values were used to calculate the absolute 16S rRNA gene copy number per milliliter, which served as a proxy for bacterial cell concentration. Because the 16S rRNA gene copy number per genome varies among bacterial taxa, bacterial loads are reported as gene copies per milliliter rather than as absolute cell counts.

2) Real-time PCR

Real-time PCR assays were performed using template dilutions and no-template controls (NTCs) to ensure specificity and monitor potential contamination. Amplification targeted the V4 hypervariable region of the 16S rRNA gene using the 515F (5′-GTGCCAGCMGCCGCGGTAA-3′) and 806R (5′-GGACTACHVGGGTWTCTAAT-3′) primers [3].

Reactions were conducted in triplicate on a QuantStudio™ 1 Real-Time PCR System (Applied Biosystems, Foster City, CA, USA) using 96-well optical plates. Each 20-μL reaction contained amfiSure qGreen Q-PCR Master Mix (Q5600; GenDEPOT), 1 μM of each primer, and 1 μL of the sample DNA. The thermal cycling profile consisted of an initial denaturation step at 94°C for 2 min, followed by 40 cycles of 94°C for 15 s (denaturation), 58.5°C for 30 s (annealing), and 72°C for 30 s (elongation). Absolute quantification was performed using the standard curve method. Post-

amplification, the PCR products were purified using the QIAquick PCR Purification Kit (Qiagen, Valencia, CA, USA) in accordance with the manufacturer's instructions to remove primers, nucleotides, and enzymes

Table S1 - Standardized inhaler technique checklist for pressurized metered-dose inhalers (pMDIs) and Respimat® device

STEP	INHALER TECHNIQUE ASSESSMENT
**1**	Hold inhaler upright
**2**	Shake inhaler vigorously 2–3 times before use (pMDI only)
**3**	Exhale gently and completely away from mouthpiece
**4**	Tilt head slightly back with chin raised
**5**	Place mouthpiece between teeth and seal lips firmly
**6**	Actuate device while inhaling slowly and deeply
**7**	Hold breath for up to 10 s (or as long as comfortable)
**8**	Exhale gently through nose or mouth
**9**	Wait 30–60 s before repeating maneuver (if second dose required)
**10**	Rinse mouth with water and spit out

Table S2 - Taxonomy annotations at the phylum and genus levels for the 10 amplicon sequence variants (ASVs) identified as potential contaminants using the decontam R package

ASV	Phylum	Genus
**bc55e65da76c7933738f8039646b180b**	*Actinomycetota*	*Lawsonella*
**5d00cbe3935c4108e757b343ea2d0bbb**	*Pseudomonadota*	*Unknown (Family Comamonadaceae)*
**6af639d0dae3106c01c2c21d78fe2a5a**	*Pseudomonadota*	*Enhydrobacter*
**d3e6abb7bc8ab6eaf44304970cc77d0d**	*Bacillota*	*Oribacterium*
**247e0e8582764b66fafa750566a12617**	*Actinomycetota*	*Micrococcus*
**5e830064cff492857877521f66c96657**	*Pseudomonadota*	*Acinetobacter*
**e5116f86e7fb0e8f11d9729eb5d343d0**	*Bacillota*	*Oribacterium*
**0eb8e1f6faf9bb43adf758f99c0baa3c**	*Actinomycetota*	*Rhodoluna*
**ff30fa6a77c90231992918d9882c87fb**	*Patescibacteria*	*Incertae_Sedis*
**13f6c6451f4d63d9f16fa6c91a8d9031**	*Patescibacteria*	*Candidatus_Saccharimonas*

Ten ASVs (total frequency=1,080 reads) were flagged as potential contaminants by the *decontam* R package [4] based on the combined method. This method identifies contaminating sequences based on their DNA concentration and prevalence across both inhaler samples and negative controls, utilizing probability thresholds of 0.1 for both parameters. ASVs identified as potential contaminants by this procedure were subsequently removed from the inhaler samples before the downstream analyses. Taxa annotations were determined using a naïve Bayes classifier [5] trained on the SILVA bacterial database [6] (SSU Ref NR 99, 138.2 dataset).

Table S3 – Distribution of the most frequent bacterial phyla across inhaler samples (pooled pMDI and Respimat® devices, n=10)

Phylum	Number of Reads	Mean	Median	IQR
Pseudomonadota	107,392	35.37	26.25	25.23
Bacillota	90,283	29.73	28.14	16.21
Bacteroidota	60,330	19.87	17.16	17.47
Actinomycetota	32,453	10.69	10.34	5.34
Fusobacteriota	10,596	3.49	2.94	2.69
Others	2,578	0.85	0.00	0.23

Note: Taxonomic classifications for the phyla *Proteobacteria* and *Firmicutes* were updated to *Pseudomonadota* and *Bacillota*, respectively, in accordance with the SILVA database [6] (release 138.2, SSU Ref NR 99).

Abbreviations: IQR, interquartile range.

Table S4 – Distribution of the most frequent bacterial genera across inhaler samples (considering pooled pMDI and Respimat® devices, n=10)

Genus	Number of Reads	Mean	Median	IQR
Haemophilus	46,010	15.15	11.44	14.56
Neisseria	42,496	14.00	18.02	14.37
Prevotella	36,408	11.99	6.28	14.12
Streptococcus	32,365	10.66	9.17	6.61
Veillonella	29,312	9.65	6.53	7.03
Rothia	24,238	7.98	9.10	6.43
Staphylococcus	13,198	4.35	0.29	0.37
Fusobacterium	7,514	2.47	2.05	1.86
Pseudomonas	6,485	2.14	0.33	1.26
Actinobacillus	6,087	2.00	0.19	3.02
Porphyromonas	5,580	1.84	0.99	1.06
Hoylesella	3,956	1.30	0.72	1.11
Gemella	3,553	1.17	1.03	1.07
Corynebacterium	3,263	1.07	0.88	0.85
Granulicatella	3,096	1.02	0.72	0.39
Others	40,071	13.20	0.00	0.09

Abbreviations: IQR, interquartile range.

Table S5 – Relative abundance of predominant bacterial phyla stratified by inhaler device (pMDI vs. Respimat®)

Phylum	pMDI (n=6)			Respimat® (n=4)			p-value	Adjusted p-value
Phylum	Mean	Median	IQR	Mean	Median	IQR		
Pseudomonadota	39.13	37.40	26.65	29.28	24.31	8.35	0.831	ns
Bacillota	30.78	30.49	16.75	28.04	24.75	11.36	1.000	ns
Bacteroidota	19.05	16.15	16.30	21.20	21.50	12.32	0.67	ns
Actinomycetota	7.49	7.86	2.06	15.86	15.66	5.31	**0.011**	**0.053**
Fusobacteriota	2.92	2.30	2.40	4.42	3.54	1.93	0.522	ns

P-values were determined using the Mann-Whitney U test. Adjusted p-values were calculated using the Benjamini-Hochberg (BH) method to control the false discovery rate (FDR) [7].

Abbreviations: ns, non-significant ($p > 0.05$).

Table S6 Relative abundance of predominant bacterial genera stratified by inhaler device (pMDI vs. Respimat®)

Genus	pMDI (n=6)			Respimat® (n=4)			p-value	Adjusted p-value
Genus	Mean	Median	IQR	Mean	Median	IQR		
Haemophilus	19.32	16.33	12.23	8.42	6.80	8.90	0.286	ns
Neisseria	11.88	10.62	14.75	17.42	19.19	4.41	0.201	ns
Prevotella	10.71	5.08	11.56	14.07	13.18	13.54	0.286	ns
Streptococcus	9.78	8.80	5.63	12.09	11.01	7.00	0.522	ns
Veillonella	10.26	4.17	7.33	8.67	9.03	3.30	0.522	ns
Rothia	4.94	4.65	4.56	12.91	12.59	4.09	0.033	ns
Staphylococcus	6.55	0.32	0.23	0.78	0.16	0.86	0.517	ns
Fusobacterium	2.44	1.91	1.81	2.53	2.15	1.06	0.831	ns
Pseudomonas	2.72	0.33	0.48	1.19	0.76	1.89	0.829	ns
Actinobacillus	2.84	1.64	4.61	0.65	0.06	0.85	0.271	ns
Porphyromonas	2.45	0.99	3.41	0.85	0.84	1.00	0.670	ns
Hoylesella	1.88	1.17	2.23	0.36	0.34	0.70	**0.087**	**ns**
Gemella	1.02	1.09	0.92	1.42	0.74	1.68	0.831	ns
Corynebacterium	0.95	0.42	0.65	1.27	1.22	0.36	0.136	ns
Granulicatella	0.79	0.63	0.19	1.39	1.00	0.50	0.088	ns

P-values were determined using the Mann-Whitney U test. Adjusted p-values were calculated using the Benjamini-Hochberg (BH) method to control the false discovery rate (FDR) [7].

Abbreviations: ns, non-significant ($p > 0.05$).

Figure S1 – Relative abundance of dominant bacterial phyla and genera in paired pMDI and Respimat® inhaler samples used by the same participants (n=2) over a 30-day period.

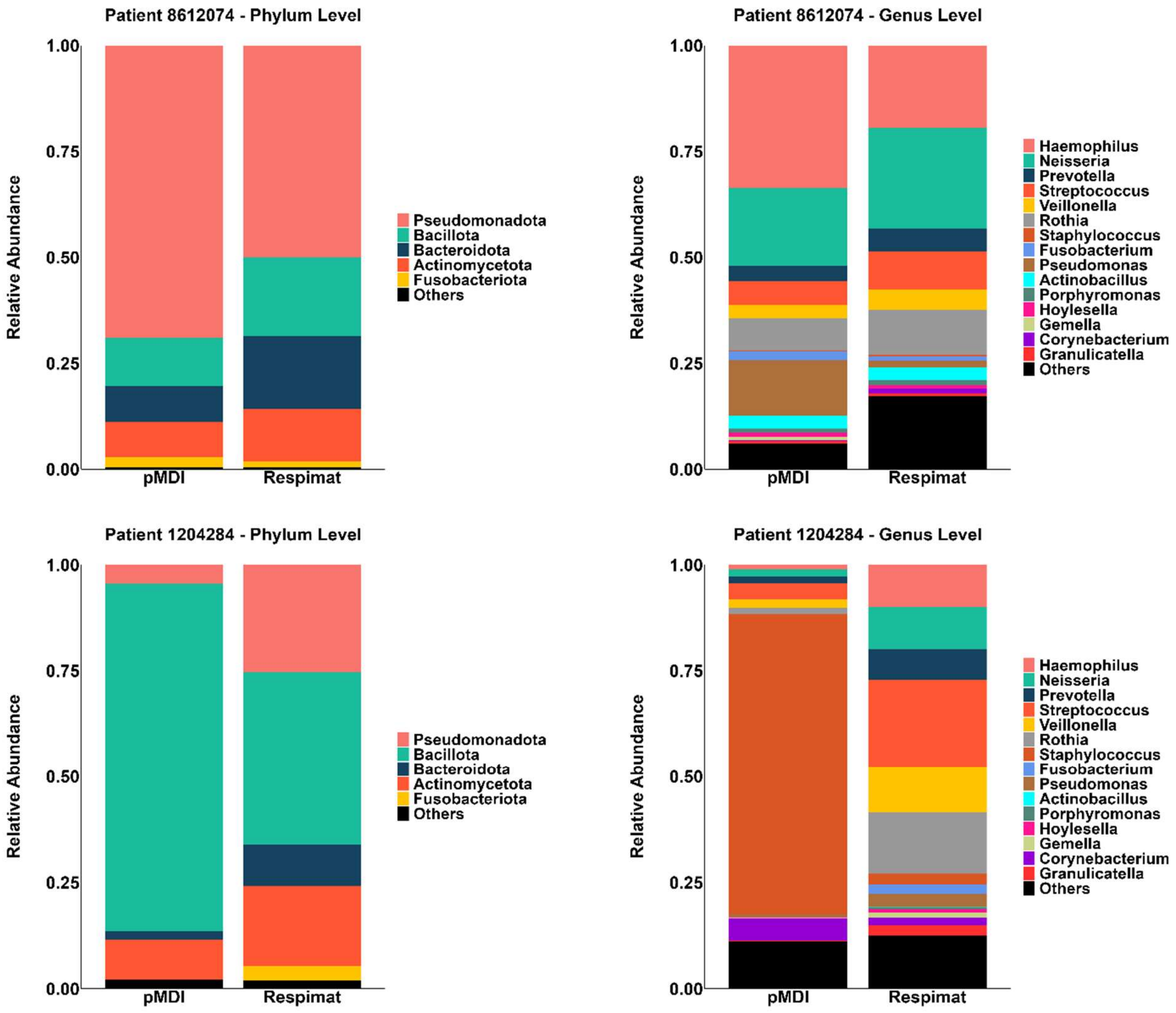

Data are shown for participants 8612074 (top panels) and 1204284 (bottom panels).

Table S7. Within-patient comparison of selected bacterial genera between pMDI and Respimat samples

Genus	pMDI	Respimat®	Effect size			Fisher exact test	
Genus	Reads (%)	Reads (%)	Fold change[1]	$\log_2$FC[1]	OR[2]	Crude p-value	BH-adjusted p-value
**Patient 8612074**							
Pseudomonas	4,635 (13.151)	357 (1.510)	8.71	3.12	9.87	<0.001	<0.001
Staphylococcus	56 (0.159)	77 (0.326)	0.49	-1.04	0.49	<0.001	<0.001
Haemophilus	11,872 (33.685)	4,607 (19.490)	1.73	0.79	2.10	<0.001	<0.001
Streptococcus	1,966 (5.578)	2,155 (9.117)	0.61	-0.71	0.59	<0.001	<0.001
**Patient 1204284**							
Pseudomonas	86 (0.514)	1,022 (3.018)	0.17	-2.55	0.17	<0.001	<0.001
Staphylococcus	11,855 (70.903)	829 (2.448)	28.96	4.86	96.86	<0.001	<0.001
Haemophilus	177 (1.059)	3,355 (9.907)	0.11	-3.23	0.10	<0.001	<0.001
Streptococcus	641 (3.834)	7,021 (20.732)	0.18	-2.44	0.15	<0.001	<0.001

Values in the pMDI and Respimat® columns represent genus-specific read counts, with relative abundances (%) shown in parentheses.

[1]Fold change was calculated as the relative abundance in pMDI divided by that in Respimat. A fold change >1 and a positive $\log_2$ FC indicate higher relative abundance in pMDI, whereas a fold change <1 and a negative $\log_2$ FC indicate higher relative abundance in Respimat.

[2]Odds ratio (OR) represents the odds that a sequencing read was assigned to the genus of interest in pMDI compared with Respimat. Crude p-values were determined using two-sided Fisher's exact tests. Multiple comparisons across the four prespecified genera were corrected within each patient using the Benjamini–Hochberg false discovery rate procedure. Benjamini–Hochberg-adjusted p-values <0.001 are reported as <0.001.

Abbreviations: OR, odds ratio; BH, Benjamini–Hochberg; $\log_2$ FC, $\log_2$ fold change.

To explore differences in sequencing-read composition, a separate 2 × 2 contingency table was generated for each genus within each patient. Rows represented inhaler devices (pMDI and Respimat®), and columns represented reads assigned to the target genus versus all remaining reads. Remaining reads were calculated by subtracting the genus-specific count from the total sequencing reads in that sample, thereby comparing the proportion of target-genus reads between devices. For example, regarding to the *Staphylococcus* abundance in patient 8612074, the pMDI sample contained 56 *Staphylococcus* reads out of 35,244 total reads (35,188 non-*Staphylococcus* reads). The Respimat® sample contained 77 *Staphylococcus* reads out of 23,638 total reads (23,561 non-*Staphylococcus* reads), forming the 2 × 2 table for the analysis. Two-sided Fisher's exact tests were applied to each table. This approach was selected because it directly evaluates associations within a 2 × 2 frameworks without relying on large-sample approximations for expected cell frequencies. Odds ratios (ORs) served as effect-size measures, where an OR >1 indicates greater odds of assignment to the pMDI sample and an OR <1 indicates greater odds in the Respimat sample.

Because four prespecified genera were evaluated per patient, correction for multiple testing was applied to the four Fisher's exact test p-values. Adjusted p-values were calculated separately for each patient using the Benjamini–Hochberg procedure. Both crude and adjusted p-values are reported, with values below 0.001 designated as <0.001.

References

1. Galazzo, G.; van Best, N.; Benedikter, B.J.; Janssen, K.; Bervoets, L.; Driessen, C.; Oomen, M.; Lucchesi, M.; van Eijck, P.H.; Becker, H.E.F.; et al. How to Count Our Microbes? The Effect of Different Quantitative Microbiome Profiling Approaches. *Front Cell Infect Microbiol* **2020**, *10*, 403. doi:10.3389/fcimb.2020.00403.
2. Yang, W.H.; Heithoff, D.M.; Aziz, P.V.; Sperandio, M.; Nizet, V.; Mahan, M.J.; Marth, J.D. Recurrent infection progressively disables host protection against intestinal inflammation. *Science* **2017**, *358*. doi:10.1126/science.aao5610.
3. Caporaso, J.G.; Lauber, C.L.; Walters, W.A.; Berg-Lyons, D.; Lozupone, C.A.; Turnbaugh, P.J.; Fierer, N.; Knight, R. Global patterns of 16S rRNA diversity at a depth of millions of sequences per sample. *Proc Natl Acad Sci U S A* **2011**, *108 Suppl 1*, 4516–4522. doi:10.1073/pnas.1000080107.
4. Davis, N.M.; Proctor, D.M.; Holmes, S.P.; Relman, D.A.; Callahan, B.J. Simple statistical identification and removal of contaminant sequences in marker-gene and metagenomics data. *Microbiome* **2018**, *6*, 226. doi:10.1186/s40168-018-0605-2.
5. Bokulich, N.A.; Kaehler, B.D.; Rideout, J.R.; Dillon, M.; Bolyen, E.; Knight, R.; Huttley, G.A.; Caporaso, J.G. Optimizing taxonomic classification of marker-gene amplicon sequences with QIIME 2's q2-feature-classifier plugin. *Microbiome* **2018**, *6*, 90. doi:10.1186/s40168-018-0470-z.
6. Quast, C.; Pruesse, E.; Yilmaz, P.; Gerken, J.; Schweer, T.; Yarza, P.; Peplies, J.; Glockner, F.O. The SILVA ribosomal RNA gene database project: improved data processing and web-based tools. *Nucleic Acids Res* **2013**, *41*, D590–596. doi:10.1093/nar/gks1219.
7. Benjamini, Y.; Hochberg, Y. Controlling the False Discovery Rate: A Practical and Powerful Approach to Multiple Testing. *Journal of the Royal Statistical Society. Series B (Methodological)* **1995**, *57*, 289–300. https://doi.org/10.1111/j.2517-6161.1995.tb02031.x.
